# DOT-SLAM: A Stereo Visual Simultaneous Localization and Mapping (SLAM) System with Dynamic Object Tracking Based on Graph Optimization

**DOI:** 10.3390/s24144676

**Published:** 2024-07-18

**Authors:** Yuan Zhu, Hao An, Huaide Wang, Ruidong Xu, Zhipeng Sun, Ke Lu

**Affiliations:** 1 School of Automotive Studies, Tongji University, Shanghai 201800, China; yuan.zhu@tongji.edu.cn (Y.Z.); hao_an@tongji.edu.cn (H.A.); huaide_wang@tongji.edu.cn (H.W.); rd_xu@tongji.edu.cn (R.X.); 2 Nanchang Automotive Institute of Intelligence & New Energy, Tongji University, Nanchang 330052, China; sunzhipeng@naiine.com

**Keywords:** stereo visual SLAM, dynamic scene, graph optimization, object tracking, non-holonomic constraint

## Abstract

Most visual simultaneous localization and mapping (SLAM) systems are based on the assumption of a static environment in autonomous vehicles. However, when dynamic objects, particularly vehicles, occupy a large portion of the image, the localization accuracy of the system decreases significantly. To mitigate this challenge, this paper unveils DOT-SLAM, a novel stereo visual SLAM system that integrates dynamic object tracking through graph optimization. By integrating dynamic object pose estimation into the SLAM system, the system can effectively utilize both foreground and background points for ego vehicle localization and obtain a static feature points map. To rectify the inaccuracies in depth estimation from stereo disparity directly on the foreground points of dynamic objects due to their self-similarity characteristics, a coarse-to-fine depth estimation method based on camera–road plane geometry is presented. This method uses rough depth to guide fine stereo matching, thereby obtaining the 3 dimensions (3D)spatial positions of feature points on dynamic objects. Subsequently, by establishing constraints on the dynamic object’s pose using the road plane and non-holonomic constraints (NHCs) of the vehicle, reducing the initial pose uncertainty of dynamic objects leads to more accurate dynamic object initialization. Finally, by considering foreground points, background points, the local road plane, the ego vehicle pose, and dynamic object poses as optimization nodes, through the establishment and joint optimization of a nonlinear model based on graph optimization, accurate six degrees of freedom (DoFs) pose estimations are obtained for both the ego vehicle and dynamic objects. Experimental validation on the KITTI-360 dataset demonstrates that DOT-SLAM effectively utilizes features from the background and dynamic objects in the environment, resulting in more accurate vehicle trajectory estimation and a static environment map. Results obtained from a real-world dataset test reinforce the effectiveness.

## 1. Introduction

As the development of intelligent vehicles advances, high-precision localization has become essential for autonomous driving. Stereo vision simultaneous localization and mapping (SLAM), which utilizes stereo cameras, provides accurate localization for intelligent vehicles [1]. Unlike localization systems that rely on Global Navigation Satellite System (GNSS), visual SLAM can deliver precise and reliable localization and mapping in environments without GNSS signals [1,2]. Additionally, compared to LiDAR SLAM, stereo cameras are not only more cost-effective and easier to install, but they also furnish scale information about the environment and offer rich texture and color details [3].

Visual SLAM systems [4,5,6] typically assume that the environment is static or quasi-static, meaning there are no or only a few dynamic objects present. Based on this assumption, these systems can achieve accurate localization and environmental mapping in low-dynamic scenes, such as indoors. However, in road scenes, there are numerous dynamic objects, especially vehicles, which may cover a substantial part of the image and severely affect the precision of ego vehicle localization and mapping [7].

To address the aforementioned issues, the intuitive approach is to eliminate as many features as possible from dynamic objects in the scene and rely exclusively on features from the static environment for pose estimation and map construction. There are two methods for removing dynamic objects. The first is to treat features on dynamic objects as outliers using methods such as random sample consensus (RANSAC) [4,5], robust kernel [8], and geometric consistency functions [9], which allows for more accurate computation of the pose transformations between camera frames. While this approach reduces the influence of dynamic objects in predominantly static scenes, it leads to a significant decrease in pose estimation accuracy in scenes with many dynamic objects or where such objects cover a substantial part of the image, making it less suitable for road scenes. The other method involves using semantics [10,11,12] and object detection [13,14] to detect and categorize objects, determine their motion states based on prior class, and remove features from dynamic objects. This can prevent interference from dynamic objects for the localization system. However, in highly dynamic scenes, directly removing dynamic features can result in a scarcity of static features and uneven feature distribution, which also leads to decreased accuracy and stability in pose estimation [15].

In recent years, dynamic SLAM systems that are coupled with dynamic object tracking have gained widespread attention. These methods [16,17,18,19,20] leverage semantic segmentation or object detection to detect and track dynamic objects, allowing foreground features to also be used for ego pose estimation. Subsequently, bundle adjustment (BA) is implemented with ego pose, dynamic objects, and feature points. These approaches have demonstrated great precision and stability in dynamic scenes [21]. However, in the dynamic scenes typical of intelligent vehicles, where most of the dynamic objects are surrounding vehicles, issues such as dynamic object initialization and dynamic object depth estimation have not been deeply explored. For dynamic object initialization, in ClusterSLAM [16] and DynaSLAMII [18], the poses of dynamic objects are initialized with the center of mass of the 3D points and with the identity matrix. While this method is reasonable for the position, initializing the orientation with an identity matrix can lead to subsequent dynamic object movements that do not conform to their kinematic constraints, introducing inevitable errors into the system. In ClusterVO [17], the pose of dynamic objects is initialized using the center and the three principal orthogonal directions of the point clouds belonging to the object as the translational and rotational components; this method relies on the completeness of the point cloud distribution. Additionally, due to the self-similarity of the local features of vehicles, directly using stereo disparity in DynaSLAMII [18] to obtain their three-dimensional structure can easily lead to mismatches, introducing depth errors for sparse feature points. In VDO-SLAM [20], using depth maps increases the front-end computational complexity and affects the real-time operational performance of the system.

This paper introduces a stereo visual SLAM system in this paper, named DOT-SLAM, which combines graph optimization and dynamic object tracking. To address the uncertainty in the initialization poses of dynamic objects, the proposed system utilizes constraints from the local road plane and the non-holonomic constraints (NHCs) of vehicles to recover the vehicle’s orientation, reducing the uncertainty in the initialization pose and providing an accurate initial value for dynamic object tracking and optimization processes. To solve the problem of large depth estimation errors for foreground points due to the self-similarity of dynamic objects when using stereo disparity directly, a coarse-to-fine depth estimation method is proposed. This method obtains a rough depth of dynamic objects using camera–road plane geometry, which then guides fine object-level stereo matching to obtain accurate foreground point depth. Ultimately, a nonlinear optimization model utilizing graph optimization is developed that jointly optimizes foreground feature points, background feature points, the local road plane, the ego vehicle pose, and dynamic object poses. The proposed system results in an accurate ego vehicle pose, dynamic object poses, and environmental map, enhancing the precision and stability of the system’s localization and mapping in dynamic environments.

This paper makes four key contributions:

1. A graph optimization framework that is tightly coupled is introduced. This framework employs rigid body motion to establish reprojection errors for features associated with dynamic objects and performs joint optimization of the vehicle pose, dynamic object poses, local road plane, and visual feature points as optimization nodes.
2. A method for initializing the pose of dynamic objects is introduced, utilizing local road plane constraints and non-holonomic constraints. This method significantly reduces uncertainty and enhances the accuracy of dynamic objects’ initial poses.
3. A multi-scale depth estimation method for dynamic objects is presented. It starts with a coarse initial pose derived from the camera–road plane geometry, which then guides refined stereo matching to ascertain the 3D spatial locations of feature points on dynamic objects.
4. A unified SLAM system is introduced that is capable of creating a globally consistent map of the static environment. The system’s performance is validated through one public dataset and real vehicle experiments, demonstrating superior localization accuracy compared to state VSLAM and VISLAM in highly dynamic scenes for the vehicle.

This paper is structured into the following sections: Section 2 reviews background research related to the topic. Section 3 introduces the notations and pose representations of dynamic objects. Section 4 gives an overview of the entire system and its individual modules. Section 5 details the experimental setup, results, and their analysis. Finally, Section 6 presents the conclusions.

## 2. Related Work

### 2.1. Visual SLAM Systems in Dynamic Scenes

Visual SLAM methods designed for dynamic scenes primarily achieve localization by filtering out dynamic points, categorizing these methods broadly into two main categories.

The first category is dynamic SLAM without prior-known dynamic object information. As the system lacks information about dynamic objects, it needs to segment static and dynamic features. One approach utilizes epipolar geometry to differentiate between static and dynamic features. Constraints were built using epipolar lines [22], reprojection errors [23,24], and Delaunay triangulation [25]. Kundu et al. [22] detected dynamic features by applying constraints from epipolar geometry and flow vector bounds, further refined through Bayesian filtering. In [23,24], reprojection errors were used to categorize features as either static or dynamic, based on the distance of reprojection. Dai et al. [25] employed the Delaunay triangulation algorithm to create a graph structure for map points, leveraging the connectivity of map points to separate dynamic objects from the static background, thus reducing the impact of moving objects on pose estimation. Other methods involve segmenting dynamic targets based on 2D optical flow and 3D scene flow, where optical flow and scene flow represent the motion fields in 2D and 3D spaces, respectively, reflecting the movement of dynamic objects. Klappstein et al. [26] segmented moving objects based on the extent to which they violated the expected optical flow. In another study [27], by incorporating the estimated pose transformations into the current frame, predictions were made for the previous frame’s image. The residuals between this predicted image and the camera image were then analyzed to identify dynamic objects. Yin et al. [15] presented a method that combines scene flow and inertial Inertial Measurement Unit (IMU) to detect dynamic features. Similar to [15], Song et al. [28] rejected features from dynamic objects by leveraging pose priors estimated by the IMU preintegration. These methods do not rely on prior information about dynamic objects and offer high real-time performance. However, in road scenes where dynamic objects dominate, these methods struggle to distinguish dynamic objects, leading to a significant decrease in the accuracy of pose estimation and making them less applicable to road environments.

The second category involves dynamic SLAM that couples with deep learning. Deep learning networks can provide semantic masks or bounding boxes from object detection within images, offering prior information about dynamic objects for constructing dynamic SLAM. This prior information about dynamic objects provides object categories but requires further integration with geometric methods such as motion consistency [11,12] and multi-view geometry [10,29] or optical flow techniques [30] to determine the motion state of the prior objects. DS-SLAM [12] introduced a method that combined semantic segmentation with motion consistency checks for dynamic feature detection, thus mitigating the impact of dynamic features on the system. DGS-SLAM [11] introduced a dynamic object detection module using a multinomial residual model. This module segmented motion in the scene by integrating motion residuals from neighboring frames with potential motion data from semantic segmentation. DynaSLAM [29] rejected dynamic objects by integrating semantic masks with multi-view geometry and reconstructing the static background. Blitz-SLAM [7] processed features along semantic boundaries by combining semantic masks with depth images, removing blurry noise blocks, and establishing an accurate point cloud map. Since semantic segmentation algorithms were computationally intensive, to enhance system real-time performance, Ballester et al. [31] and Singh et al. [32] did not require semantic segmentation in every frame. Instead, they combined multi-view geometry or feature flow to propagate semantics in intermediate frames, thus improving the system’s real-time performance. Meanwhile, DetectSLAM [13] and Dynamic-SLAM [14] used bounding boxes provided by object detection algorithms to identify the boundaries of potential moving objects but could not obtain the complete contours of moving targets, necessitating further differentiation between moving objects and the background. These methods utilize prior information provided by deep learning networks, combined with information from geometric or optical flow, to accurately identify dynamic objects. However, removing features from dynamic objects can lead to poorer feature distribution in road scenes, which also adversely affects the accuracy of localization [33].

### 2.2. Coupled Visual SLAM and Dynamic Object Tracking

To better address the interference of dynamic objects on SLAM systems, dynamic SLAMs that incorporate multi-object tracking have been proposed. Depending on how the system integrates both components, these systems are typically categorized as either loosely coupled or tightly coupled.

Loosely coupled dynamic SLAM and dynamic object tracking independently perform SLAM and track moving objects. DOT [31] employed multi-view geometry to follow the trajectory of dynamic objects, avoiding the segmentation of all frames in the sequence and thus enhancing the robustness and accuracy of the system in dynamic environments. The main drawback of this method is that its accuracy is highly dependent on the accuracy of the camera pose estimation. PLDS-SLAM [34] demonstrated how integrating point and line features within a dynamic SLAM context helps the system better handle urban environments where static and dynamic objects coexist. This system shows a loosely coupled integration where multiple object tracking (MOT) is used to track dynamic line features (such as those on moving vehicles), which are then processed differently from static features during SLAM computations. Hong et al. [35] correlated objects detected in different frames, which were identified by projecting their 3D bounding boxes into the bird’s-eye view. Tracking these objects to analyze their motion states allowed for distinguishing dynamic objects from static ones. These methods enhance the accuracy of dynamic object detection through multi-frame tracking, but the main drawback is that their accuracy is highly correlated with that of the camera pose estimation. RLD-SLAM [36] utilized information from the IMU to track dynamic objects, thereby enhancing the robustness of SLAM in highly dynamic environments. Similarly, DGM-VINS [37] adopted a method that leverages IMU data for dynamic object tracking rather than for camera pose estimation, which helps avoid coupling between the DOT and camera pose estimation. However, due to the constrained motion of vehicles, system initialization and ensuring observability are challenging aspects for VI-SLAM [38].

Tiny-coupled dynamic SLAM and dynamic object tracking enhance estimation accuracy by merging the poses of dynamic objects and ego motion into a single framework for simultaneous solutions. Research [39,40] has shown that the problems of SLAM and multi-object tracking are mutually beneficial. Li et al. [41] proposed a method for dynamic targets such as vehicles using 2D detection boxes, combined with the proposed viewpoint classification to generate 3D detection boxes for vehicles, which are then jointly optimized with camera poses in the back-end. CubeSLAM [42] is an object-level SLAM. It generated 3D boxes of objects using 2D detection boxes and vanishing points, tracking these boxes using semantic segmentation. These methods utilize prior information to recover the 3D spatial information of targets from 2D boxes, requiring accurate 2D detection data and heavily depending on prior information. ClusterSLAM [16] clustered point clouds of dynamic objects using rigid body motion to represent their movement without needing prior information about dynamic targets. In the back-end, it used factor graph optimization to correct the shape and trajectory of dynamic objects. ClusterVO [17], an improvement on ClusterSLAM, introduced a heterogeneous conditional random field (CRF) clustering approach for clustering and tracking dynamic objects, addressing inaccuracies in segmentation. DynaSLAMII [18] innovatively incorporated object tracking into the visual SLAM pipeline using deep learning, optimizing both scene understanding and state estimation in environments characterized by frequent dynamics. These methods do not require prior information about dynamic targets but tend to simplify the initialization of dynamic object poses using identity matrices or point cloud distributions. MOTSLAM [43] and VIMOT [44] detected 3D bounding boxes of dynamic objects using deep learning algorithms, representing the initial poses of dynamically observed targets as 3D bounding boxes. It is important to note that learning-based methods are limited in generation and challenging to adapt to complex autonomous driving scenes [45]. VDO-SLAM [20] used global dense optical flow to track dynamic feature points and performed the joint optimization of ego pose, dynamic object pose, and spatial points in the back-end, achieving good results. However, the entire system requires instance segmentation, global optical flow, and depth maps as inputs, leading to a heavy computational load at the front-end.

Due to the constraints imposed by both the environment and their kinematics, vehicles have limited freedom in their spatial movements. Therefore, these dynamic targets require precise initial pose estimation. The incorrect initialization of poses can lead to subsequent pose estimations that no longer conform to their kinematic constraints, introducing additional errors. Additionally, due to the self-similarity of local features on vehicles, directly using stereo disparity to derive their three-dimensional structure can easily lead to mismatches, resulting in depth errors for sparse feature points. In response to these challenges, this paper designs a tightly-coupled dynamic object tracking SLAM system specifically for autonomous driving scenes. For the initialization of dynamic object poses, the system extensively incorporates the vehicle’s kinematic constraints and the environmental constraints acting on the vehicle, thereby reducing the uncertainty of the dynamic objects’ poses. The system uses the coarse depth information provided by the camera–road plane geometry to guide precise stereo matching, enabling the determination of the 3D spatial positions of feature points on dynamic objects.

## 3. Preliminaries

### 3.1. Notation

Initially, the notations used throughout the paper are defined. ${\left(\cdot \right)}_{w}$ stands for the world frame, ${\left(\cdot \right)}_{c}$ stands for the camera frame, and ${\left(\cdot \right)}_{D}$ stands for the body frame of dynamic objects. The matrix representing the transformation from the world frame to the $k^{\mathrm{th}}$ camera frame is as follows:

(1) $$\left.T_{w}^{c_{k}}=\left[\begin{array}{cc} R_{w}^{c_{k}} & t_{w}^{c_{k}}\\ 0^{T} & 1 \end{array}\right]\in \mathrm{SE}\left(3\right)\right|\;R_{w}^{c_{k}}\in \mathrm{SO}\left(3\right),t_{w}^{c_{k}}\in R^{3},$$

where $R_{w}^{c_{k}}$ is a rotation matrix, $t_{w}^{c_{k}}$ is a translation vector, SE(3) is the group of 3D rigid body transformations, including rotations and translations, and SO(3) is the group of 3D rotations. By applying the transformation matrix $T_{w}^{c_{k}}$, 3D landmarks $P_{w}$ are projected from the world frame into the $k^{\mathrm{th}}$ camera frame, as follows: $P_{c_{k}}={\left(T_{w}^{c_{k}}P_{w}^{\prime }\right)}_{\left[1:3\right]}=R_{w}^{c_{k}}P_{w}+t_{w}^{c_{k}}$, where $P^{\prime }$ denotes the homogeneous version of P. K is the intrinsic matrix of the camera; the parameters of this intrinsic matrix need to be obtained in advance through calibration. Local planes of the road are generally represented in the Hesse form (HF), with the plane equation being $\pi ={\left[\begin{array}{cc} n^{T} & d \end{array}\right]}^{T}$, where n is the unit vector, with ∥n∥ = 1; *d* denotes the distance from the plane to the origin of the coordinate system. Since the HF uses four parameters to represent a plane, which is overparameterized, the closest point (CP) [46] is used to represent the plane during back-end optimization, as follows: Π=dn, where Π is the road plane.

### 3.2. Pose Representations of Dynamic Objects and Features

**Figure 1:**
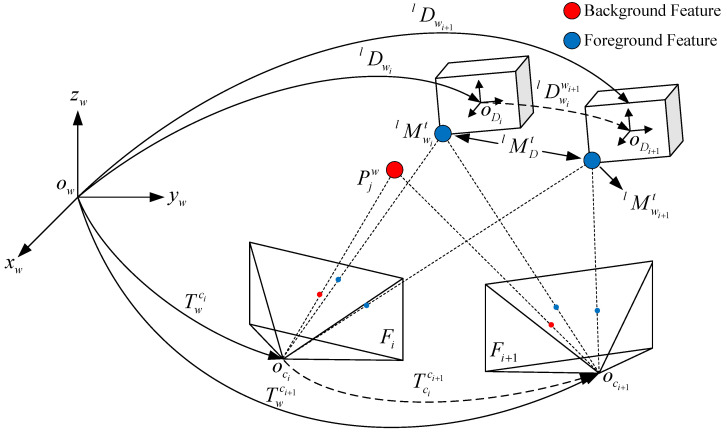
The pose representation of a dynamic object. Cubes represent the same dynamic object in different frames, solid lines are the pose transformations in the world frame, dashed lines are transformations between camera frames, and dotted lines originating from the camera optical center.

In road scenes, dynamic objects are other moving vehicles, and their motion can be represented by rigid body motion. In Figure 1, the red points represent static features, i.e., background features, while the blue points denote features from dynamic objects, i.e., foreground features. Cubes represent the same dynamic object in different camera frames. The pose transformations of the camera and dynamic objects in the world frame are indicated by solid lines, while the pose transformations between frames are represented by dashed lines. Dwil∈SE(3) represents the pose of the $l^{\mathrm{th}}$ dynamic object in the world coordinate in the $i^{\mathrm{th}}$ frame. Dwiwi+1l∈SE(3) represents the transformation of a dynamic object from the camera frame $i^{\mathrm{th}}$ to ${\left(i+1\right)}^{\mathrm{th}}$ in the world frame. Given the poses of the dynamic object in camera frames $i^{\mathrm{th}}$ and ${\left(i+1\right)}^{\mathrm{th}}$, the pose transformation of the dynamic object between consecutive frames can be expressed as follows:

(2) Dwiwi+1l=Dwi+1lDwil−1.

Next is the transformation of dynamic feature points between frames. First, let MDtl∈R3 represent the spatial position of the $t^{\mathrm{th}}$ dynamic point from the $l^{\mathrm{th}}$ dynamic object in the body frame of the dynamic object. The position of this dynamic feature point in the world frame in the $i^{\mathrm{th}}$ camera frames can be expressed as follows:

(3) Mwitl=Dwil−1MDitl,

where Mwitl and MDitl represent the spatial positions of the $t^{\mathrm{th}}$ dynamic point of the $l^{\mathrm{th}}$ dynamic object observed by the $i^{\mathrm{th}}$ camera frame within the world frame and the object’s body frame. By combining Equations (2) and (3), the following can be obtained:

(4) Mwi+1tl=Dwi+1l−1MDi+1tl=Dwil−1Dwi+1wilMDi+1tl.

Since the dynamic object is a rigid body, the spatial position of the dynamic point in its reference object coordinate system is fixed and can be expressed as follows:

(5) MDi+1tl=MwilMwitl=Dwi+1lMwi+1l.

By substituting Equation (5) into Equation (4), the following can be obtained:

(6) Mwi+1tl=Dwil−1Dwi+1wilDwilMwitl.

Using Equation (6), the transformation relationship of dynamic feature points between consecutive frames can be obtained. The three transformation matrices on the right side of Equation (6) are defined as a single transformation matrix Dwi+1wi. Equation (6) can be expressed as follows:

(7) Mwi+jtwil  =Dwi+jwi lMwitl

If the motion state of the dynamic object is taken as a state variable of the system, the reprojection error of feature points on the dynamic object can be constructed to constrain the ego vehicle pose estimation.

## 4. Proposed System

### 4.1. System Overview

**Figure 2:**
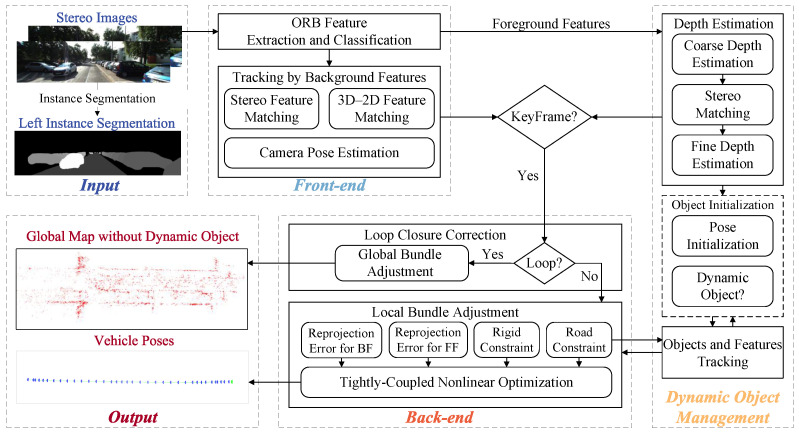
The pipeline of the proposed system. The inputs are stereo images and instance segmentation from the left camera’s images with instances of dynamic objects and masks of road. The outputs are the vehicle poses and the global map. The system consists of three parts, namely front-end, dynamic object management, and back-end.

Figure 2 depicts the pipeline of the proposed system, which includes three core components: the front-end, dynamic object management, and the back-end. The system takes stereo images and instance segmentation from the images of the left camera, with inputs consisting of instances of dynamic objects and road masks obtained from an instance segmentation network [47]. At the first component, oriented FAST and rotated BRIEF (ORB) feature extraction is performed, and the pose transformation between frames is calculated based on the background features. In dynamic object management, the first step is to perform a coarse-to-fine depth estimation of dynamic objects and the foreground points on these objects to obtain their spatial information. Then, historical dynamic objects are associated with instances in the current frame. For associated dynamic objects, the inter-frame tracking of foreground points is estimated using optical flow. If a new dynamic object is detected, it is decided that the current frame is a keyframe. The accurate global pose information of the vehicle can be initialized using the constraints between the road and the vehicle, as well as the vehicle’s kinematic constraints. Local bundle adjustment (BA) and global bundle adjustment form the backbone of the back-end. In the local BA, two types of reprojection errors are designed for dynamic and static feature points, respectively. Following previous works [48,49], the ground feature points are used to estimate the local road plane, which will be used for initializing the pose of dynamic objects. Utilizing these three constraints, a nonlinear optimization is constructed to simultaneously optimize the spatial positions of feature points, the local road plane, the ego vehicle pose, and the dynamic object poses. Global BA is executed when the system detects a loop closure, globally optimizing the vehicle trajectories and the environment map in the system to correct accumulated pose drift.

### 4.2. Front-End

The front-end follows the same process as feature-based visual SLAM [4], where the input stereo images are processed in sequence through feature extraction, feature matching between stereo images, feature matching between inter-frames, and pose estimation between inter-frames. Initially, image pyramids are constructed in both stereo images, with each level divided into several 60 × 60 patches, and, in each patch, feature points and descriptors are extracted using the ORB method [4], resulting in uniformly distributed features. Then, based on the input left image instance segmentation, the extracted features are classified into the following two categories according to the instance segmenting: background features and foreground features. It is important to note that, in the front-end, only background features are used for self-vehicle pose estimation, while the extracted foreground points are sent to the dynamic object management module for further processing. These static points are then stereo matched to obtain their spatial information and associated with feature points in the local map using 3D–2D correspondence. Based on the matching results, the current frame’s pose is computed using perspective-n-point (PnP). The PnP estimates the camera’s position and orientation by solving for the transformation from known 3D points to their corresponding 2D image points.

### 4.3. Dynamic Object Management

**Figure 3:**
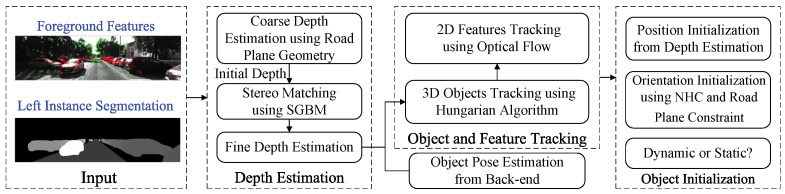
The pipeline of dynamic object management module. The red points are the foreground features and the green points are the background features in the upper left image.

As shown in Figure 3, the dynamic object management module consists of the following three submodules: depth estimation, object-level and feature-level tracking of dynamic objects, and object initialization. First, the depth of dynamic objects is estimated using stereo disparity. A coarse depth estimation method based on camera–road plane geometry and a fine depth estimation method based on semi-global block matching (SGBM) [50] are proposed. The coarse depth guides the SGBM stereo disparity search range, resulting in more accurate depth estimation. Different tracking algorithms are designed for object-level and feature-level tracking. The Hungarian algorithm [51] is used for object-level matching. Object-level tracking guides feature-level tracking by using the result of optical flow to ensure the accuracy of feature-level tracking. For unassociated instances, the dynamic object initialization module performs pose initialization and motion state determination for new instances. The initial position information can be obtained from depth estimation. Using constraints between the road and vehicle kinematic constraints, the vehicle’s accurate pose can be initialized. Lastly, scene flow is used to determine the motion state of the instance; only the pose of moving objects is estimated in the back-end, while stationary objects are treated as static features to estimate the ego vehicle pose.

#### 4.3.1. Depth Estimation

For each dynamic object, in order to achieve stable inter-frame tracking, in addition to the foreground features extracted in the front-end, ordinary pixels are selected at fixed intervals in a 5 × 5 grid, following the method from VDO [20]. These pixel points, together with the foreground feature points, will be used to track the inter-frame motion of dynamic objects, and are collectively referred to as dynamic feature points in the following text. For these dynamic feature points, stereo disparity is used to estimate their depth to recover their spatial position. Here, the SGBM algorithm is employed to calculate the stereo disparity. This algorithm uses semi-global matching cost aggregation, which only considers the matching cost of local blocks, achieving high computational efficiency and matching accuracy.

**Figure 4:**
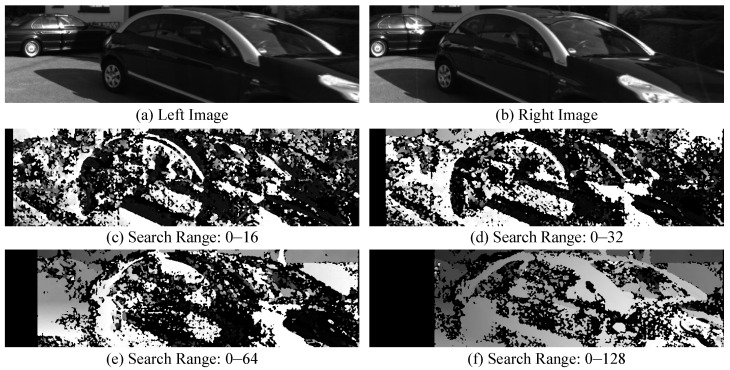
The results of SGBM with different initial values of the disparity search range.

The SGBM algorithm is sensitive to the initial values of the disparity search range, as using different initial values can significantly impact depth computation. Figure 4 shows the disparity result with different initial values. As the initial value of the disparity search range increases, the disparity maps become more continuous and the disparity computation improves. However, a larger initial disparity value also means reduced computational efficiency, making the selection of an appropriate initial value crucial.

**Figure 5:**
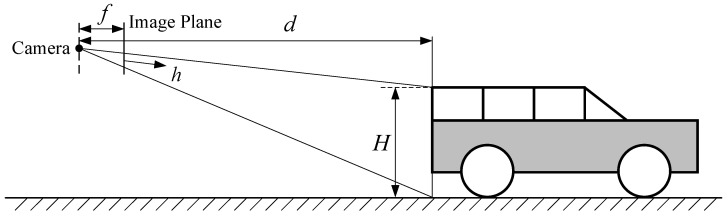
Depth estimation using camera–road plane geometry.

Due to the significant variation in the spatial positions of dynamic objects, the best approach is to determine a rough depth for each dynamic object and allocate an independent initial value for the disparity search range for each one. Here, road plane geometry is used to determine the initial depth of dynamic objects. As shown in Figure 5, with a camera focal length *f*, a front vehicle model height *H*, a projection height *h* in the image plane, and a distance *d* from the front vehicle to the camera, the depth *d* of the front vehicle can be approximately expressed as follows:

(8) $$d=\frac{f_{y}H}{h},$$

where $f_{y}$ is the focal length in the *y* direction of the camera measured in pixels. Based on the depth value, the rough disparity can be calculated as follows:

(9) $$disp=\frac{f_{x}hb}{f_{y}H},$$

where disp is the disparity of the dynamic object, $f_{x}$ is is the focal length in the *x* direction of the camera measured in pixels, and *b* is the baseline of the stereo camera.

The specific calculation process for selecting the initial value of the disparity search range is shown in Algorithm 1. Lines 1–3 initialize the variables needed for the algorithm; lines 4–17 iterate over the pixels of the input instance segmentation image, saving the minimum and maximum row coordinates for each instance; lines 18–22 use Equation (9) to calculate the rough disparity. Since the SGBM algorithm requires the disparity search range to be a multiple of 16, it is converted to a multiple of 16 in line 21. After obtaining the initial value of the disparity search range, the image needs to be sliced into patches corresponding to each dynamic object, as shown in Figure 4. Then, the SGBM algorithm is used to calculate the disparity map between the left and right image patches. According to the disparity map, the accurate depth of the dynamic feature points is computed, which allows the determination of their spatial positions.    
**Algorithm 1:** Calculation of stereo disparity search range
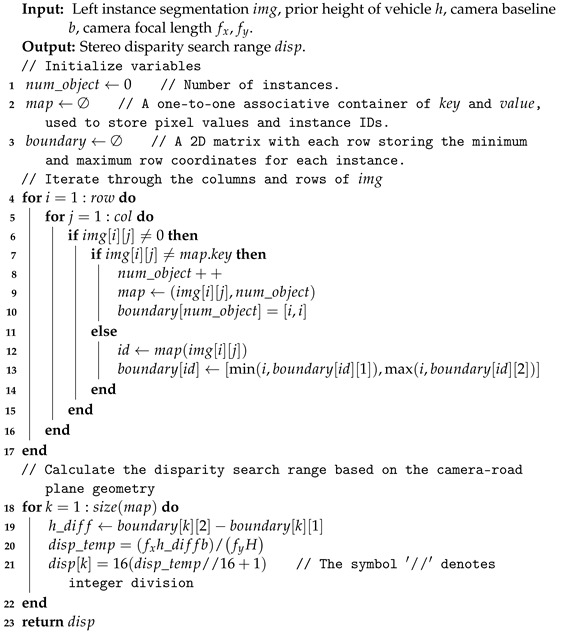


#### 4.3.2. Object and Feature Tracking

**Figure 6:**
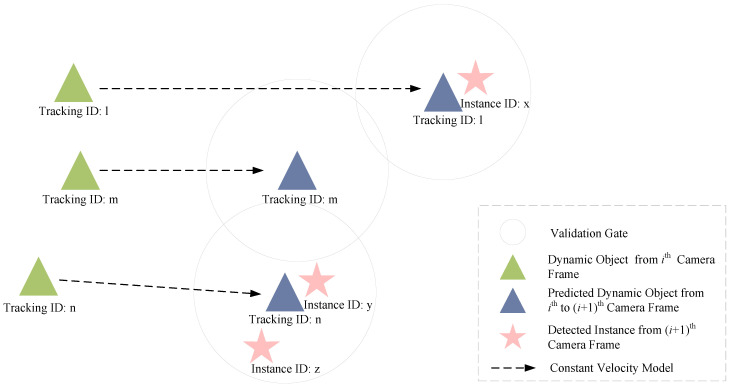
Tracking-by-detection method for object-level tracking. Dynamic objects from the $i^{\mathrm{th}}$ camera frame, represented by green triangles, are predicted in the ${\left(i+1\right)}^{\mathrm{th}}$ camera frame as blue triangles, using a constant velocity model. Association gates are then established around these predicted dynamic objects, within which instances detected in the ${\left(i+1\right)}^{\mathrm{th}}$ camera frame are potentially associated with the predicted dynamic objects.

In the tracking module, both object-level and feature-level tracking are required. Object-level tracking adopts the tracking-by-detection method from multi-object tracking [51], matching instances in consecutive frames to establish correspondences, as shown in Figure 6. First, the dynamic objects tracked in the $i^{\mathrm{th}}$ frame are extrapolated to the ${\left(i+1\right)}^{\mathrm{th}}$ frame using a constant velocity model. Then, instances in the ${\left(i+1\right)}^{\mathrm{th}}$ frame are searched within a fixed-size validation gate centered on the predicted object. For each predicted object, there are two possible situations. The first situation is that no instance falls within the gate, indicating that the dynamic object is no longer in the camera’s field of view or has become occluded. Using the constant velocity model, the system extrapolates the object’s position for the next three frames, and if no instance is associated within three consecutive frames, the object is removed from the tracked objects. The second situation is that at least one instance falls within the validation gate. The distance from the object to all instances is calculated. Once the association results for all targets are obtained, the Hungarian algorithm is used to match each object with the most suitable instance, achieving a globally optimal tracking result. Unassociated instances are sent to the subsequent object initialization module for pose and motion state initialization.

**Figure 7:**
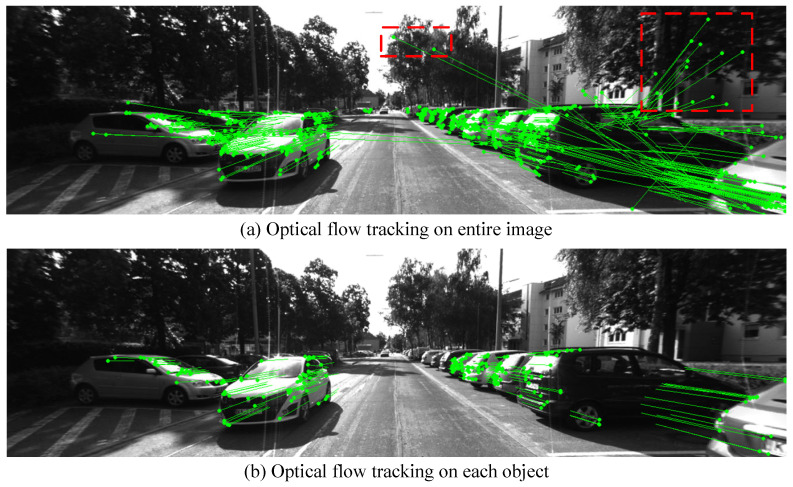
Feature-level tracking by optical flow. Incorrect tracking is marked in red boxes.

After completing object-level tracking, feature-level tracking of the feature points on the objects is required. Because of the movement of dynamic objects, the expansion of the search area and significant viewpoints changes, making feature matching difficult. By leveraging the object-level tracking results, feature-level tracking can be guided. The images of consecutive frames are divided into patches according to the objects, and for each dynamic object there is a pair of patches. For dynamic feature point tracking, the optical flow method is applied. This local optical flow method reduces the computational load compared to the global optical flow method and avoids some erroneous associations caused by changes in lighting. As shown in Figure 7, the top figure is the tracking result using the global optical flow on the entire image, and the bottom figure is the result using the proposed method of optical flow tracking for each object. It can be seen that feature-level tracking guided by object-level tracking effectively reduces the number of incorrect associations and improves the accuracy of feature point tracking.

#### 4.3.3. Object Initialization

**Figure 8:**
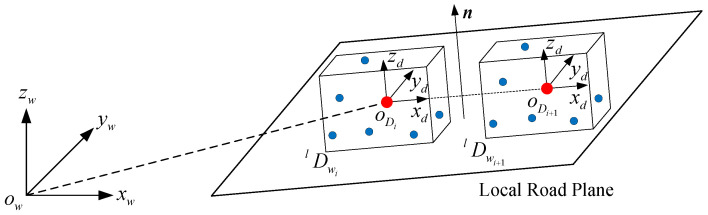
The initial position and orientation of dynamic objects. The blue points are the features in dynamic objects. The cubes represent the positions of the vehicle in consecutive frames, with the vehicle moving closely along the local road plane.

Instances that are not successfully associated in the object tracking module are considered to be new dynamic objects, and their positions and poses need to be initialized. In Figure 8, the blue points represent the dynamic feature points of the dynamic object, and the centroid of the dynamic feature points is represented by the red point. Determining the initial position is relatively simple; the centroid of all feature points in the dynamic object is used as its initial position. Once the centroid of the dynamic object is determined during initialization, the centroid position remains unchanged in subsequent tracking, meaning that the origin of the reference object coordinate system does not change during the motion of the dynamic object. This ensures that the positions of the feature points in the dynamic object’s coordinate system remain fixed.

Since the pose of the dynamic object cannot be directly obtained during initialization, some methods [16,18] directly initialize the pose as an identity matrix. Such a simple initialization method will result in the dynamic object’s motion not satisfying the kinematic constraints of the vehicle during subsequent tracking, i.e., the vehicle’s lateral and vertical velocities are both zero, introducing inevitable errors in the motion estimation of the dynamic object. To address this issue, it is necessary to determine the directions of the three coordinate axes centered at the centroid. For the direction of the *z* axis, refer to the constraints between the vehicle and the road plane, as mentioned in [48,49]. Since the dynamic object moves along the road plane, the normal *n* of the road plane remains parallel to the *z*-axis direction of the vehicle. For the direction of the *x* axis, it can be obtained by calculating the translation vector $t_{w_{i}}^{w_{i+1}}$ between consecutive frames. Addressing the challenge of tracking initial objects without motion information, a zero-velocity model is employed, initializing the speed of the dynamic object to zero and temporarily ignoring its orientation. During associations between consecutive frames, a relatively large association gate is designed. This allows correct instances in the current frame to fall into the association gate of the zero-velocity dynamic object initialized in the previous frame. The robustness of the Hungarian algorithm is relied upon for correct association. In cases of fast-moving dynamic objects or those with large angular velocities, it is acceptable for the dynamic object to be incorrectly associated. In such cases, the instance in the current frame is initialized as a zero-velocity dynamic object and participates in the association of instances in the next frame. Once the zero-velocity dynamic object is associated with an instance, it can be initialized according to the following method. Due to the non-holonomic constraints of the vehicle, meaning the vehicle has only lateral velocity, the *x* axis direction should remain parallel to the translation vector. $v_{y}$ and $v_{z}$ are unit vectors parallel to the *y* axis and *z* axis, respectively. $v_{y}$ and $v_{z}$ can be expressed as follows:

(10) $$\left\{\begin{array}{c} v_{x}=\frac{t_{w_{i}}^{w_{i+1}}}{∥t_{w_{i}}^{w_{i+1}}∥}\\ v_{z}=n \end{array}\right..$$

It is important to note that the obtained *z* axis and *x* axis may not be perpendicular. Therefore, the *z*-axis direction needs to be adjusted slightly to ensure it remains perpendicular to the *x* axis. The adjustment of the z-axis direction consists of two steps. First, calculate the projection of vector $v_{z}$ onto vector $v_{x}$, as shown below:

(11) $${\mathrm{proj}}_{v_{y}}\left(v_{z}\right)=\frac{v_{x}v_{z}}{{∥v_{x}∥}^{2}}v_{x},$$

where ${\mathrm{proj}}_{v_{y}}\left(v_{z}\right)$ is the projection vector of $v_{z}$ onto $v_{x}$. Then, the orthogonalized vector is calculated as follows:

(12) $${\tilde{v}}_{z}=\frac{v_{z}-{\mathrm{proj}}_{v_{x}}\left(v_{z}\right)}{∥v_{z}-{\mathrm{proj}}_{v_{x}}\left(v_{z}\right)∥},$$

where ${\tilde{v}}_{z}$ represents the adjusted unit vector. According to the properties of the Cartesian coordinate system, the *y*-axis direction of the dynamic object can be calculated using the cross-product of vectors, as shown below:

(13) $$v_{y}=v_{x}\times v_{z},$$

where $v_{y}$ is the *y*-axis direction of the object and × is the cross-product of the vectors. After obtaining the directions of the three axes of the dynamic object, the initial pose $R_{D}\in \mathrm{SO}\left(3\right)$ of the dynamic object can be determined, as shown below:

(14) $$R_{D}=\left[\begin{array}{ccc} v_{x} & v_{y} & {\tilde{v}}_{z} \end{array}\right].$$

The initial pose $T_{D}\in \mathrm{SE}\left(3\right)$ of the dynamic object can be expressed as follows:

(15) $$T_{D}=\left[\begin{array}{cc} R_{D} & M_{D}\\ 0^{T} & 1 \end{array}\right],$$

where $M_{D}$ is the centroid of the dynamic object.

Directly treating all prior dynamic objects as dynamic objects not only increases computational complexity but also affects the accuracy of self-vehicle pose estimation. Therefore, accurately determining whether prior dynamic classes are in motion is crucial for tracking dynamic objects. Scene flow is a method commonly employed to differentiate between the states (dynamic or static) of objects in the scenes. The spatial position of dynamic objects changes over time, and, if considered as a collection of spatial points, all these points move in the same direction; therefore, it appears that the spatial points are flowing through space, hence the term “scene flow”. In contrast, the spatial positions of static objects do not change over time, so there is no scene flow for them. Therefore, scene flow can help ascertain whether previously identified dynamic objects are actively moving. The scene flow $\Delta M\in R^{3}$ of a dynamic object from the $i^{\mathrm{th}}$ frame to the ${\left(i+1\right)}^{\mathrm{th}}$ frame can be expressed as follows:

(16) ΔM=1m∑t=1mMwi+1tl−Mwitl,

where *m* is the number of tracked feature points in the $i^{\mathrm{th}}$ dynamic object. It is important to note that, due to the errors of depth estimation, the scene flow of a stationary dynamic object will not be exactly zero. Therefore, a threshold (set to 0.15 in this paper) is required to determine whether an object is stationary or in motion.

### 4.4. Back-End

Upon detection of keyframes in the front-end, they are subsequently transmitted to the back-end. Here, a process of local BA is executed to optimize all keyframes within the sliding window. The size of the sliding window in the proposed system is not fixed; all co-visible keyframes of the current keyframe are searched and added to the sliding window. At the same time, a loop check is performed to assess if the current keyframe forms a loop closure with previously stored keyframes in the map. If the conditions for loop closure are met, a global BA is carried out to correct for loop closure.

#### 4.4.1. Local Bundle Adjustment

**Figure 9:**
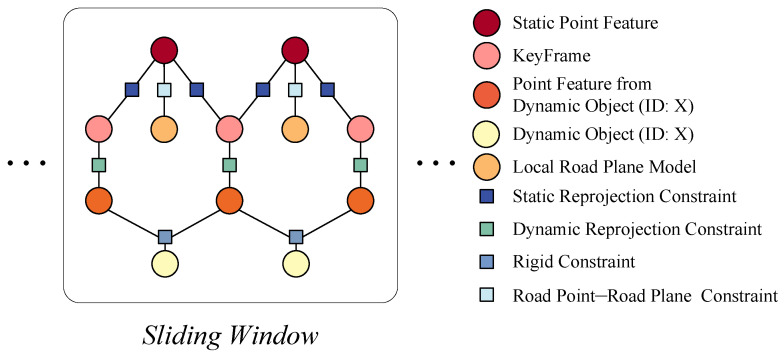
Factor graph of the nonlinear optimization in local bundle adjustment.

In local bundle adjustment (LBA), optimization is required for the poses of keyframes and dynamic objects, the positions of dynamic and static feature points, and the local road plane. Figure 9 shows the various optimization variables and their corresponding four types of constraints: the reprojection constraint from static feature points, the reprojection constraint from dynamic feature points, the rigid body constraint between dynamic feature points and dynamic objects, and the constraint between road feature points and the local road plane. Constraints are established between the vehicle camera poses and feature points, allowing for the adjustment of the camera poses and the spatial positions of the feature points. This ensures that the rays reflected from each feature point converge at the camera center after adjustment. This process optimizes the poses of multiple cameras and the spatial coordinates of the landmarks. Since dynamic objects are in motion, these moving feature points cannot directly establish constraints with the vehicle. If the motion state of the dynamic object is known, the motion of the feature points on the dynamic object can be compensated for, enabling these dynamic feature points to establish constraints with the vehicle camera poses. This requires simultaneous optimization of the camera poses and the motion states of the dynamic objects to achieve minimal reprojection error. The error between the local road plane and road feature points does not constrain the camera pose. The fitted plane from road points is primarily used for the initialization of dynamic objects. The error functions are formulated as least squares and iteratively solved using the Gauss–Newton method by the G^2^o solver [52], with the maximum number of iterations set to 10. Based on the above constraints, the corresponding loss functions are established and iteratively optimized. In local bundle adjustment, the objective is to minimize the following loss function:

(17) $$E_{LBA}=\sum_{i,j}{∥e_{1}^{i,j}∥}_{\Sigma_{1}^{-1}}+\sum_{i,l,t}{∥e_{2}^{i,l,t}∥}_{\Sigma_{2}^{-1}}+\sum_{i,l,t}{∥e_{3}^{i,l,t}∥}_{\Sigma_{3}^{-1}}+\sum_{k,j}{∥e_{4}^{k,j}∥}_{\Sigma_{4}^{-1}},$$

where $e_{1}$ is the reprojection error from static feature points, $e_{2}$ is the reprojection error from dynamic feature points, $e_{3}$ is the error constructed between dynamic feature points and dynamic objects, and $e_{4}$ is the error constructed between road points and the local road plane. $\Sigma_{1}^{-1}$, $\Sigma_{2}^{-1}$, $\Sigma_{3}^{-1}$, and $\Sigma_{4}^{-1}$ represent the information matrices corresponding to the four types of errors.

$e_{1}$ is the reprojection error from the static feature points. By constructing the reprojection error from 3D map points to image feature points in the keyframe, 3D spatial points are projected onto the imaging plane of the keyframe. Adjustments are made to both the pose of the keyframe and the positions of the map points to minimize the distance between the projected position and the associated feature points. The error is represented as follows:

(18) $$e_{1}^{i_{j}}=e\left(T_{w}^{c_{i}},P_{j}^{\prime }\right)=u_{j}-\frac{1}{s_{j}}KT_{w}^{c_{i}}{P_{j}}^{\prime },$$

where $T_{w}^{c_{i}}$ is the pose of the $i^{\mathrm{th}}$ keyframe, $P_{j}^{\prime }$ is the homogeneous form of the $j^{\mathrm{th}}$ map point, K is the intrinsic matrix of the camera, $u_{j}$ represents the pixel coordinates of the feature point associated with the map point $P_{j}$, and $s_{j}$ represents the depth of the map point $P_{j}$. Using the pyramid level at which the feature point was extracted to represent the information matrix of the reprojection error, the information matrix $\Sigma_{1}^{-1}$ for the feature point extracted at level *n* can be expressed as follows:

(19) $$\Sigma_{1}^{-1}=\frac{1}{s^{n}p}\left[\begin{array}{cc} 1 & 0\\ 0 & 1 \end{array}\right],$$

where *p* is the standard deviation at level 0 of the image pyramid, and *s* is the scale of the image pyramid.

$e_{2}$ is the reprojection error from dynamic feature points. For feature points on dynamic objects, the position of the dynamic object in space can be obtained by tracking its motion, and this position can be reprojected into the image. The distance between the reprojected position and the associated pixel is then calculated according to the optical flow estimation results. Through adjustments to the pose of the keyframe and the position of the dynamic feature points, the error is minimized. The error is shown as follows:

(20) e2i,l,t=eTwci,Mwitl=uitl−1sitlKTwci·Mwitl′,

where $T_{w}^{c_{i}}$ is the pose of the $i^{\mathrm{th}}$ keyframe, Mwitl′ is the homogeneous form of the $t^{\mathrm{th}}$ dynamic feature point in the $l^{\mathrm{th}}$ dynamic object, $u_{i}^{t}$ represents the pixel coordinates of the feature point associated with the feature point Mwitl′, and sitl represents the depth of the dynamic feature point Mwitl. Since the dynamic feature points are tracked using the optical flow estimation results, the information matrix $\Sigma_{2}^{-1}$ can be constructed using the photometric error, as shown below:

(21) $$\Sigma_{2}^{-1}=\frac{1}{1+|p-q|}\left[\begin{array}{cc} 1 & 0\\ 0 & 1 \end{array}\right],$$

where *p* and *q* are the pixel values of the matching pixel points in the two frames used for optical flow.

$e_{3}$ depicts the constraint error of the rigid body between the dynamic object and its dynamic feature points. Since the dynamic object is assumed to be a rigid body, the positions of the feature points on the same dynamic object are fixed in consecutive frames, which is referred to as the rigid body constraint. The following loss function can be constructed based on the rigid body constraint between the dynamic object and the dynamic feature points:

(22) e3i,l,t=eMwitl,Mwi−1tl,Dwiwi−1l       =Mwitl−Dwiwi−1l       ·Mwi−1tl,

where Mwitl and Mwi−1tl are the positions of the same feature point in two consecutive frames and Dwiwi−1l        is the motion of the dynamic object between two consecutive frames. For depth calculated using stereo disparity, the depth estimation of the feature points is inaccurate, leading to errors in their spatial information. Therefore, the information matrix $\Sigma_{3}^{-1}$ can be expressed as follows:

(23) Σ3−1=1sitl·σ100010001,

where sitl is the depth of Mwitl and σ is the standard deviation of depth estimation.

The Jacobian formulas of the error function $e_{3}$, with respect to each optimization variable, are shown below:

(24) JMwitl=−Dwi+1wil  ,

(25) JDwi+1wil  =−MwitlT⊗I,

where ⊗ is the Kronecker product.

$e_{4}$ is the error between the local road plane and road feature points. For the local road plane, instance segmentation can be used to obtain feature points on the road. These feature points are then used to fit the local road plane. In the fitting process, the error is represented by the distance from the road points to the plane, as follows:

(26) $$e_{4}^{k,j}=e\left(\Pi_{k},P_{j}\right)=∥\Pi_{k}∥-\frac{\Pi_{k}^{T}P_{j}}{∥\Pi_{k}∥},$$

where $P_{j}$ represents the feature points located on the road, $\Pi_{k}$ is the $k^{\mathrm{th}}$ road plane represented in closest point form, and $\Pi_{k}^{T}$ is the transpose of $\Pi_{k}$. The smaller the distance from the road points to the plane, the closer the road points are to the plane, and the better the local plane fits the actual road surface. The error between the local road plane and the road points mainly arises from the uncertainty in the spatial positions of the points. The construction of its information matrix $\Sigma_{4}^{-1}$ is similar to the reprojection error of static feature points and can be expressed as follows:

(27) $$\Sigma_{4}^{-1}=\frac{1}{\left(s^{n}p\right)}\left[\begin{array}{ccc} 1 & 0 & 0\\ 0 & 1 & 0\\ 0 & 0 & 1 \end{array}\right],$$

where *p* is the standard deviation at level 0 of the image pyramid, *s* is the scale of the image pyramid, and *n* is the number of levels in the pyramid.

The Jacobian formulas of the error function $e_{4}$, with respect to $\Pi_{k}^{T}$ and $P_{j}$, are shown below:

(28) $$J\left(\Pi_{k}^{T}\right)=\frac{\Pi_{k}^{T}}{∥\Pi_{k}∥}+\frac{P_{j}^{T}\Pi_{k}\Pi_{k}^{T}}{{∥\Pi_{k}∥}^{3}}-\frac{P_{j}^{T}}{∥\Pi_{k}∥},$$

(29) $$J\left(P_{j}\right)=-\frac{\Pi_{k}^{T}}{∥\Pi_{k}∥},$$

where $\Pi_{k}^{T}$ is the $k^{\mathrm{th}}$ road plane represented in closest point form, and $P_{j}$ is the road feature points.

#### 4.4.2. Loop Correction

Loop correction and LBA are conducted concurrently in the back-end. When a new keyframe is detected, the system employs a bag-of-words model, specifically DBoW2 [53], akin to visual SLAM systems [4,5], to identify potential loops with historical keyframes in the map. If a loop is confirmed, a global bundle adjustment corrects accumulated drift within the loop. Throughout global optimization, only the poses of keyframes and static feature points are adjusted to refine the static map’s accuracy.

## 5. Experiments

The evaluation of DOT-SLAM included the publicly available KITTI-360 dataset [54], as well as real-world datasets collected with actual vehicles. For comparison, the open-source visual SLAM systems ORB-SLAM2 [4] and OV^2^-SLAM [6], the visual–IMU fusion SLAM system ORB-SLAM3 [5], and the dynamic visual SLAM system DynaSLAM [18] were also tested on these datasets to evaluate the performance of DOT-SLAM. As mentioned before, DynaSLAM, a state-of-the-art system for removing dynamic objects in dynamic SLAM, uses semantic and multi-view geometry to detect moving objects and effectively filter out dynamic objects.The experimental setup utilized a computer equipped with an Intel i7-11700 CPU (Intel, Santa Clara, CA, USA) operating at 3.6 GHz, 16 GB RAM, and an NVIDIA RTX 3070 GPU (NVIDIA, Santa Clara, CA, USA) for implementing the proposed system and comparison systems. It is worth emphasizing that both systems were run on each sequence to reduce the impact of randomness in each system.

To evaluate the performance of the proposed system and the comparison systems, two metrics were used: absolute trajectory error (ATE) [55] and relative pose error (RPE) [56]. The absolute trajectory error (ATE) evaluates the global consistency of the system by comparing the root mean square error (RMSE) between the estimated trajectory and the ground truth. RPE evaluates the local accuracy across every camera frame in this paper, making it suitable for assessing system drift, including the relative translational error $t_{rel}$ and the relative rotational error $r_{rel}$. Before evaluation, it is crucial to align the coordinate systems of each system with the ground truth, and the Umeyama algorithm [57] was utilized for this purpose.

### 5.1. KITTI-360 Dataset

The KITTI-360 dataset includes nine sequences that provide ground truth for vehicle poses, with a total length of 73.7 km, including urban streets, residential areas, and highways. This dataset has various sensor data, including a stereo color camera with a resolution of 1408 × 376 pixels, operating at a frame rate of 10 Hz and with a baseline of 0.6 m. Additionally, the dataset includes data from a 64-line LiDAR and an OXTS3003 GPS/IMU unit, which provides global localization results. The fisheye cameras have a 180-degree field of view (FOV). In this experiment, calibrated stereo images and ground truth data from the dataset were utilized. It’s important to note that the ground truth was derived from OXTS measurements, laser scans, and multi-view images as inputs, through large-scale optimization, making it more accurate and reliable. Another reason for using this dataset is that KITTI-360 contains more scenes with dynamic objects, making it more suitable for evaluating the proposed system. However, this dataset has a significant issue: the provided ground truth is not completely continuous, and some images in the dataset do not have corresponding ground truth poses available, so the dataset sequences were filtered and segmented. Therefore, all sequences were processed to obtain segments with continuous ground truth poses, with the starting and ending camera frames of each sequence marked. Except for sequences 02 and 09, the other seven sequences contain dynamic scenes with dynamic objects, thus referred to as dynamic sequences. All selected segments of sequences do not contain loop closure scenes, so none of the algorithms detected loop closures or executed global bundle adjustment.

Table 1 shows the evaluation results of the proposed system and four other methods. In sequences 02 and 09, where the scenes are predominantly static with minimal dynamic objects, the proposed system achieved results similar to ORB-SLAM2 and slightly better than DynaSLAM. The static assumption-based ORB-SLAM2 and ORB-SLAM3 achieved better localization accuracy in such scenes due to the absence of dynamic objects. In these sequences, the proposed system performed similarly to ORB-SLAM2 and outperformed DynaSLAM. This is because, even though there were no dynamic vehicles, there were many stationary vehicles along the roadsides. The proposed method utilized scene flow during the dynamic object initialization phase to determine the motion status of vehicles and did not perform continuous tracking of stationary vehicles. This approach led to results similar to ORB-SLAM2. In contrast, DynaSLAM failed to fully utilize the features on stationary vehicles, resulting in poorer localization results compared to ORB-SLAM2. Specifically analyzing the results from KITTI-360 sequence 04, it was found that the proposed method had relatively high rotational errors in the initial frames. Although the average relative rotational error was small, the propagation of initial errors over time led to greater deviations from the ground truth in the absolute trajectory. This error propagation is the contributing factor to the observed discrepancy wherein the system exhibits minimal relative errors but not the minimal absolute trajectory errors.

**Figure 10:**
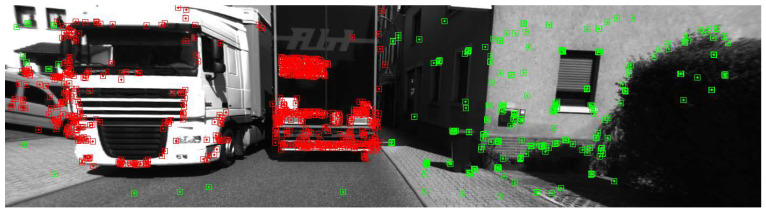
ORB features were detected in frame 4218 of KITTI-360 sequence 05. Features of dynamic objects are marked in red, while other features are marked in green.

Overall, the proposed system outperforms the two stereo visual SLAM systems, ORB-SLAM2 and OV^2^-SLAM, in most dynamic sequences. ORB-SLAM3 does not show significant improvement in localization accuracy over ORB-SLAM2 due to the short sequences and insufficient stimulation of the IMU. Consequently, the performance of ORB-SLAM3 is also inferior to the proposed system. When compared to DynaSLAM, the proposed system outperforms DynaSLAM, except in sequence 07. In sequence 07, which has fewer dynamic objects, DynaSLAM achieves the best localization results. In sequence 00, which has fewer dynamic objects and can be considered a low-dynamic scene, DynaSLAM and the proposed system exhibit similar performances. ORB-SLAM2 and ORB-SLAM3 also extract stable static feature points from all feature points, achieving good localization accuracy. In sequences 03 and 10, which have a higher number of moving objects, and sequence 05, where dynamic objects occupy a large portion of the image, the proposed system outperforms DynaSLAM in terms of both translational and rotational errors, which aligns with the previous analysis. As shown in Figure 10, when dynamic objects occupy a large portion of the image in sequence 05, removing these objects leads to poor feature distribution. Particularly, when dynamic objects are close to the ego vehicle, the removal of nearby features results in the loss of crucial points that leads to rising ambiguity when estimating translation and rotation. By combining multi-object tracking with the SLAM method, the proposed system utilizes a greater number of more evenly distributed feature points, thereby achieving better pose accuracy.

**Figure 11:**
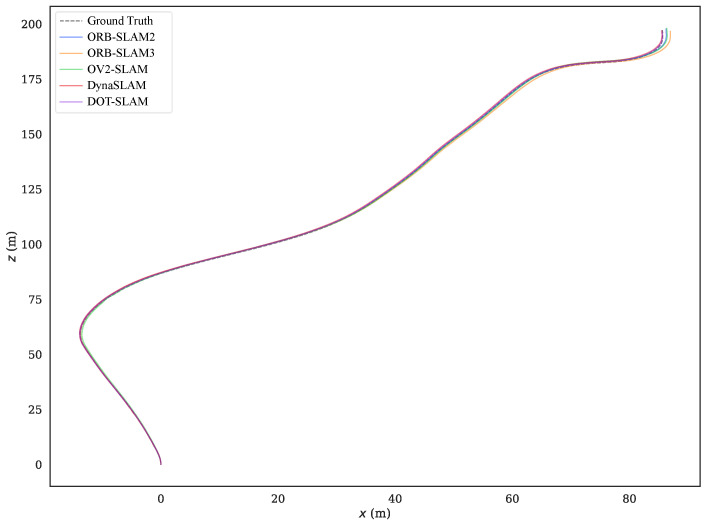
Estimated trajectories along with the ground truth for KITTI-360 sequence 05.

**Figure 12:**
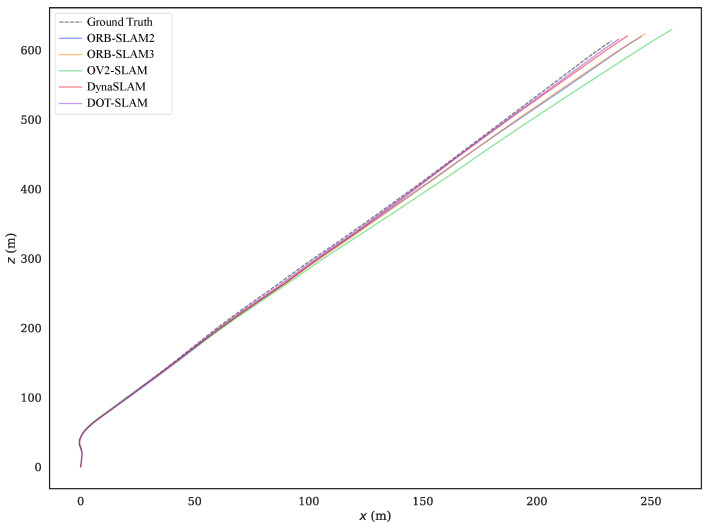
Estimated trajectories along with the ground truth for KITTI-360 sequence 10.

**Figure 13:**
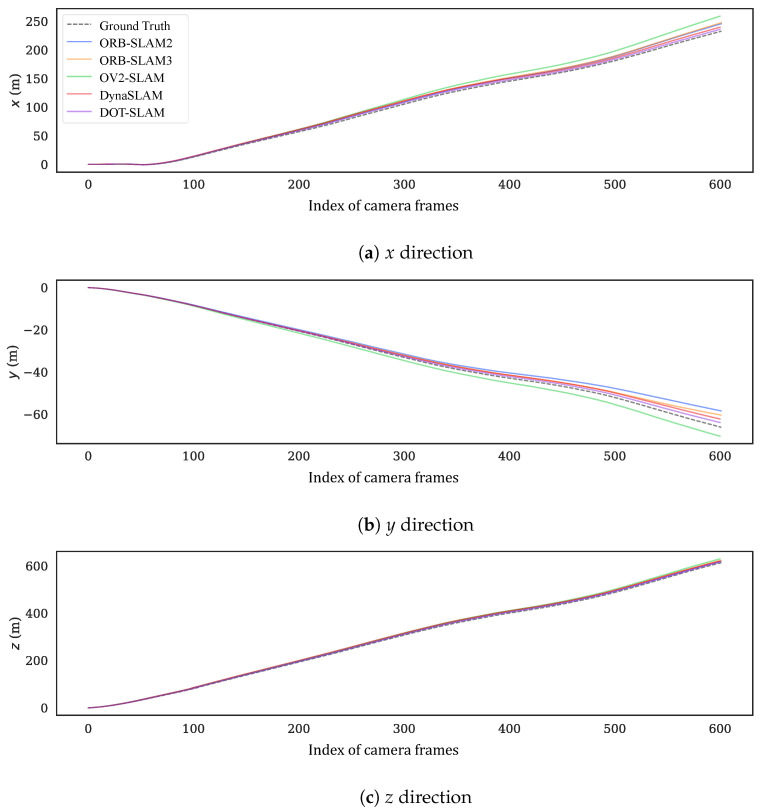
Estimated positions along with ground truth in three directions for KITTI-360 sequence 10.

To more intuitively compare the alignment of the estimated trajectories of the proposed method and the comparison methods with the ground truth, Figure 11 and Figure 12 show the estimated trajectories along with ground truth for KITTI-360 sequences 05 and 10. From these images, it can be seen that the trajectory estimated by DOT-SLAM is closer to the ground truth, which corresponds to the minimized ATE demonstrated by the proposed system, as shown in Table 1. Figure 13a–c further show the comparison of DOT-SLAM and the comparison methods with the ground truth in three directions on KITTI-360 sequence 10. It can be seen that the proposed system outperforms the other systems, with smaller errors in the lateral and longitudinal directions, bringing it into closer alignment with the ground truth. These two directions, in contrast to the vertical direction, are more affected by dynamic objects. This is because, under the static background assumption, the motion of vehicles moving in the same or opposite direction is transferred to the estimation of poses of the ego vehicle.

### 5.2. Real-World Experiments

**Figure 14:**
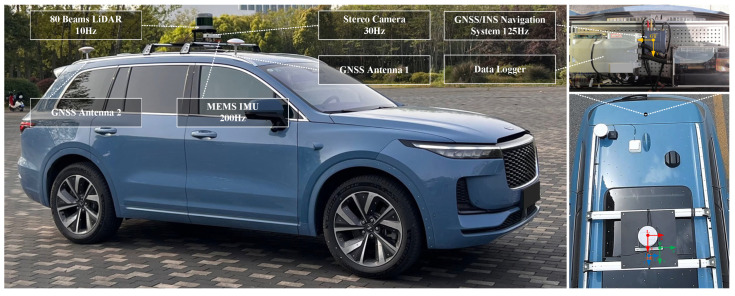
The data collection vehicle and its equipped sensors. The sub-image shows coordinate systems of different sensors: the red represents the LiDAR coordinate system, the green represents the stereo camera coordinate system, and the blue represents the IMU coordinate system.

A data collection vehicle, equipped with a stereo camera that had a baseline of 0.2 m and a resolution of 1280 × 720 at 30 Hz, was used to gather real-world scene data. The vehicle also featured an Xsens MTI-300 IMU operating at 200 Hz, a LiDAR with a 10 Hz frequency, and a Bynav GNSS/IMU unit. All sensor data were recorded by a dedicated data logger. Before conducting the experiment, the extrinsic parameters between sensors and the intrinsic parameters of the stereo cameras were obtained through calibration. The coordinate systems of the sensors are illustrated on the right side of Figure 14. In this experiment, the sensor data used included images from the calibrated stereo color camera, IMU data, and localization results from the Bynav GNSS/IMU unit. The localization results, after time synchronization and coordinate transformation, were used as ground truth to evaluate the systems. It is worth noting that, compared to the stereo camera used in KITTI-360, the collection vehicle was outfitted with a stereo camera featuring a compact 0.2 m baseline, which is more representative of real vehicle configurations. The smaller stereo disparity meant that the systems could only use feature points close to the vehicle. Among the four sequences, sequences 01, 02, and 03 were collected on campus, with fewer dynamic objects. Sequence 00 was collected on a straight road, with a higher number of dynamic objects. Since the KITTI-360 dataset used before did not have a loop-closure scene, a loop-closure sequence (sequence 01) was specifically collected, while the other three sequences did not contain loop-closure scenes.

**Figure 15:**
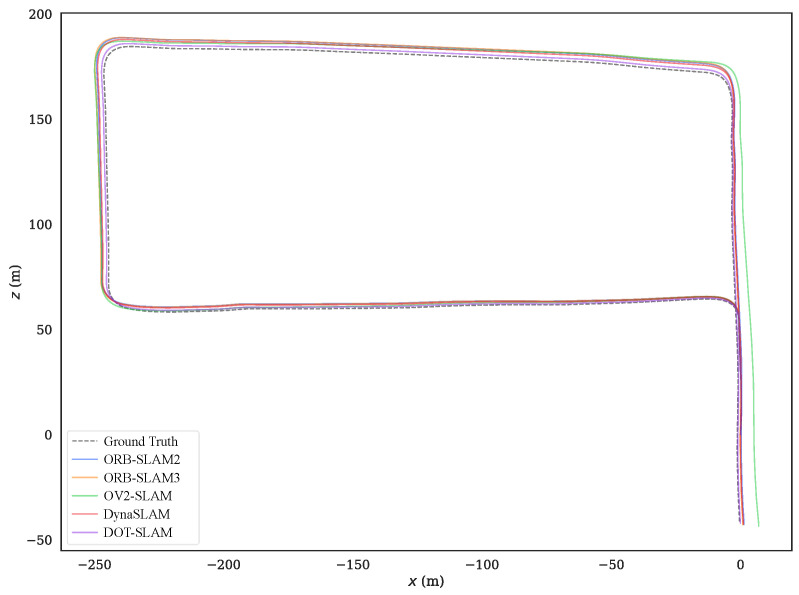
Estimated trajectories along with the ground truth for real-world sequence 01.

Table 2 displays the experimental results of DOT-SLAM and the comparison systems on the real-world dataset collected in the campus and highway scenes. OV^2^-SLAM demonstrated poor stability when detecting loop closure, while the other systems identified loop closure in sequence 01 and applied correction accordingly, as shown in Figure 15. After performing loop closure, DOT-SLAM and DynaSLAM achieved the best localization accuracy. This is because both systems used only static features for loop closure optimization, excluding dynamic features, which also allowed for the construction of a globally consistent static map. In sequences 02 and 03, which had fewer dynamic objects, the proposed system achieved better results than DynaSLAM. One of the reasons for this may be that, compared to the KITTI-360 dataset, the experiment used cameras that are more representative of those equipped on real vehicles, with a relatively smaller baseline. The smaller baseline resulted in larger depth errors from stereo matching, allowing only features closer to the vehicle to be used to reduce the impact of feature point depth errors on pose estimation. If the features on nearby dynamic objects were directly removed, it would significantly affect the number and distribution of available feature points. The proposed system tracked dynamic objects and utilized these dynamic feature points, resulting in better performance. In sequence 03, the proposed system also had the minimum relative errors, but the absolute trajectory error was higher than OV^2^-SLAM, which was similarly affected by error propagation, as mentioned above.

**Figure 16:**
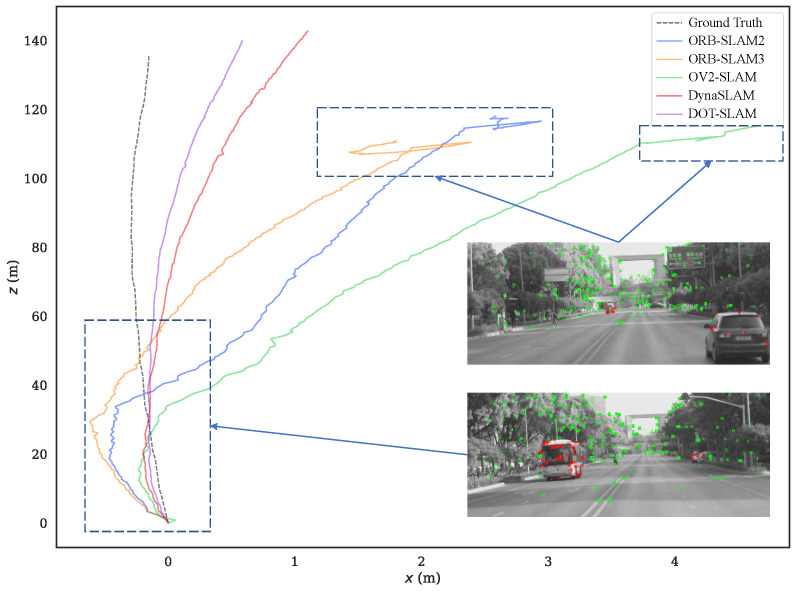
Estimated trajectories along with the ground truth for real-world sequence 00.

**Figure 17:**
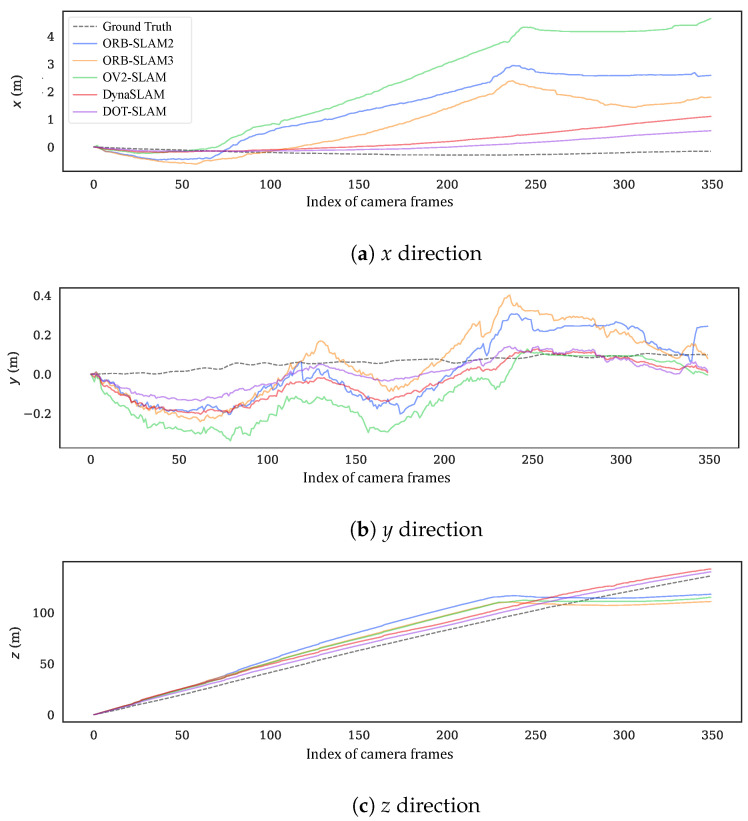
Estimated positions along with ground truth in three directions for real-world sequence 00.

Sequence 00 was collected on a straight road in front of the campus, with dynamic objects traveling in the same and opposite directions. The motion trajectories of the dynamic objects were also relatively clear, so the experimental results on this sequence were analyzed more specifically. Figure 16 shows the comparison of the trajectories of each system with the ground truth, and Figure 17a–c further illustrate the comparison of DOT-SLAM and the other systems in three directions with the ground truth. It can be seen that the other systems were significantly affected by dynamic objects except for the proposed system and DynaSLAM. At the beginning of the trajectory, as shown in the bottom right image of Figure 16, there was a moving bus in the opposite direction. The green points denote static feature points, while the red points denote dynamic feature points. The performance of the three SLAM systems relying on the static assumption was significantly impacted, and their trajectories deviated noticeably from the ground truth, while the proposed system maintained a more stable trajectory. This is because SLAM systems based on the static assumption consider the moving bus as stationary, so the feature points on the bus are treated as static feature points. In reality, these feature points moved in space, and the SLAM system incorrectly estimated the self-vehicle’s motion based on these moving feature points, resulting in erroneous pose estimation. In the latter part of the trajectory, the three SLAM systems based on the static assumption were again affected by the vehicle on the right front, causing noticeable changes in the lateral direction of the trajectory. Compared to DynaSLAM, which removes dynamic features, the proposed system had smaller lateral errors and a scale closer to the ground truth. This is because the proposed system used features on dynamic objects, making the distribution of feature points used for pose estimation more uniform. Additionally, features closer to the vehicle significantly help to improve the accuracy of both translation and rotation.

## 6. Conclusions

This work introduces a stereo visual SLAM system with dynamic object tracking based on graph optimization tailored for intelligent vehicles. The proposed system tightly coupled dynamic object tracking with the visual SLAM system, performing joint optimization of the ego vehicle pose, dynamic object poses, and feature points during BA, resulting in accurate ego vehicle pose estimation and a static map. The system fully considered the kinematic and environmental constraints of intelligent vehicles to initialize the poses of dynamic objects, improving the accuracy of dynamic object pose initialization. For dynamic object depth estimation, the system also utilized the geometric relationship between the camera and the road plane to obtain the initial depth of dynamic objects, guiding a more refined dynamic object depth estimation. Experiments using the KITTI-360 dataset demonstrated that the DOT-SLAM system could fully utilize static and dynamic features in scenes, providing more accurate vehicle trajectory estimation. Real-world data validation further proved the effectiveness of the system. In summary, the DOT-SLAM system significantly improved localization and mapping accuracy for autonomous vehicles in dynamic environments, showing higher localization precision and reliability compared to current state-of-the-art VSLAM and VISLAM systems. In future work, the proposed system will be tested in more diverse scenes. Additionally, the tracking of non-rigid moving objects will be further developed, and the accuracy of dynamic object tracking will be further optimized and evaluated.

## Figures and Tables

**Table 1:** Comparison of pose estimation in the KITTI-360 dataset $\left[t_{ate}\left(m\right),t_{rel}\left(\%\right),r_{rel}({}^{\circ}/100\;m)\right]$.

Seq.	Start/Stop Frame	DOT-SLAM			Stereo ORB-SLAM2			Stereo Inertial ORB-SLAM3			Stereo OV^2^SLAM			DynaSLAM		
		$t_{\mathrm{ate}}$	$t_{\mathrm{rel}}$	$r_{\mathrm{rel}}$	$t_{\mathrm{ate}}$	$t_{\mathrm{rel}}$	$r_{\mathrm{rel}}$	$t_{\mathrm{ate}}$	$t_{\mathrm{rel}}$	$r_{\mathrm{rel}}$	$t_{\mathrm{ate}}$	$t_{\mathrm{rel}}$	$r_{\mathrm{rel}}$	$t_{\mathrm{ate}}$	$t_{\mathrm{rel}}$	$r_{\mathrm{rel}}$
00	9693/10,220	**0.29**	0.12	0.12	0.33	**0.11**	0.13	0.46	0.18	**0.11**	1.35	0.89	0.23	0.31	0.13	0.12
02	11,432/12,944	3.58	0.46	**0.19**	**3.57**	**0.42**	0.20	4.17	0.53	0.23	4.76	0.51	0.21	3.82	0.52	0.20
03	328/900	**2.10**	**0.35**	0.18	2.47	0.38	0.22	3.48	0.56	0.25	2.40	0.78	0.45	2.25	0.36	**0.17**
04	9975/10,220	1.13	**0.33**	**0.12**	1.21	0.37	0.15	1.23	0.45	0.15	5.59	0.85	0.20	**1.11**	0.35	0.14
05	4208/4588	**0.42**	0.32	**0.20**	0.58	**0.31**	0.35	0.72	0.42	0.35	1.45	1.07	0.36	0.48	0.32	0.25
06	8805/9537	**2.49**	**0.52**	0.23	2.60	0.57	0.19	2.84	0.59	**0.18**	5.39	0.67	0.63	2.61	0.53	**0.18**
07	3/1360	4.38	0.45	**0.24**	4.63	**0.41**	0.29	4.44	0.60	0.26	8.73	0.84	0.30	**4.27**	0.45	**0.24**
09	1847/4711	6.12	0.50	0.30	6.25	0.62	**0.29**	**5.98**	0.48	0.33	6.05	**0.45**	**0.29**	6.40	0.58	0.31
10	2611/3212	**2.22**	**1.42**	0.70	3.18	1.68	0.68	3.64	2.02	**0.62**	4.82	2.45	0.70	2.48	1.58	0.72

The best results in each sequence are highlighted in bold.

**Table 2:** Comparison of pose estimation on real-world dataset $\left[t_{ate}\left(m\right),t_{rel}\left(\%\right),r_{rel}({}^{\circ}/100\;m)\right]$, with loop closure.

Seq.	DOT-SLAM			Stereo ORB-SLAM2			Stereo OV^2^-SLAM			Stereo Inertial ORB-SLAM3			DynaSLAM		
	$t_{\mathrm{ate}}$	$t_{\mathrm{rel}}$	$r_{\mathrm{rel}}$	$t_{\mathrm{ate}}$	$t_{\mathrm{rel}}$	$r_{\mathrm{rel}}$	$t_{\mathrm{ate}}$	$t_{\mathrm{rel}}$	$r_{\mathrm{rel}}$	$t_{\mathrm{ate}}$	$t_{\mathrm{rel}}$	$r_{\mathrm{rel}}$	$t_{\mathrm{ate}}$	$t_{\mathrm{rel}}$	$r_{\mathrm{rel}}$
00	**5.62**	**1.21**	**0.59**	10.01	1.52	1.09	9.04	1.59	1.25	11.32	1.61	1.28	6.25	1.43	0.64
01	**2.19**	**1.25**	**0.23**	3.56	1.52	0.24	3.77	1.74	0.33	3.74	1.85	0.26	2.93	1.39	0.43
02	**2.83**	**0.99**	**0.39**	2.94	1.03	0.45	2.84	1.32	0.40	7.01	2.49	0.56	2.92	1.04	0.43
03	3.85	**0.89**	**0.24**	3.99	0.94	0.26	**3.79**	0.93	0.29	4.70	1.06	0.33	4.04	0.98	0.26

The best results in each sequence are highlighted in bold.

## Data Availability

Publicly available datasets were analyzed in this study. These data can be found here: https://www.cvlibs.net/datasets/kitti-360/index.php, accessed on 30 January 2024.
